# Factors Associated with Gestational Diabetes Mellitus: A Meta-Analysis

**DOI:** 10.1155/2021/6692695

**Published:** 2021-05-10

**Authors:** Yu Zhang, Cheng-Ming Xiao, Yan Zhang, Qiong Chen, Xiao-Qin Zhang, Xue-Feng Li, Ru-Yue Shao, Yi-Meng Gao

**Affiliations:** ^1^Department of Obstetrics and Gynecology, People's Hospital of Chongqing Banan District, Chongqing 401320, China; ^2^Department of Obstetrics and Gynecology, Liaocheng People's Hospital and LiaochengClinical School of Shandong First Medical University, Liaocheng, Shandong 252000, China; ^3^School of Clinical Medicine, Chongqing Medical and Pharmaceutical College; Chongqing Engineering Research Center of Pharmaceutical Sciences, Shapingba District, Chongqing, 401331, China

## Abstract

Gestational diabetes mellitus (GDM) is a major public health issue, and the aim of the present study was to identify the factors associated with GDM. Databases were searched for observational studies until August 20, 2020. Pooled odds ratios (ORs) were calculated using fixed- or random-effects models. 103 studies involving 1,826,454 pregnant women were identified. Results indicated that maternal age ≥ 25 years (OR: 2.466, 95% CI: (2.121, 2.866)), prepregnancy overweight or obese (OR: 2.637, 95% CI: (1.561, 4.453)), family history of diabetes (FHD) (OR: 2.326, 95% CI: (1.904, 2.843)), history of GDM (OR: 21.137, 95% CI: (8.785, 50.858)), macrosomia (OR: 2.539, 95% CI: (1.612, 4.000)), stillbirth (OR: 2.341, 95% CI: (1.435, 3.819)), premature delivery (OR: 3.013, 95% CI: (1.569, 5.787)), and pregestational smoking (OR: 2.322, 95% CI: (1.359, 3.967)) increased the risk of GDM with all *P* < 0.05, whereas history of congenital anomaly and abortion, and HIV status showed no correlation with GDM (*P* > 0.05). Being primigravida (OR: 0.752, 95% CI: (0.698, 0.810), *P* < 0.001) reduced the risk of GDM. The factors influencing GDM included maternal age ≥ 25, prepregnancy overweight or obese, FHD, history of GDM, macrosomia, stillbirth, premature delivery, pregestational smoking, and primigravida.

## 1. Introduction

Gestational diabetes mellitus (GDM), defined as glucose intolerance of variable degree with onset or first recognition during pregnancy, is reported as one of the most common clinical complications of pregnancy [1, 2]. According to International Diabetes Federation (IDF) 2017, the prevalence of GDM is expected to be on the rise year by year [3]. Women with GDM may incur a potential risk of adverse outcomes [4, 5]. Mothers who have GDM are at risk of developing gestational hypertension and preeclampsia, at risk of suffering from caesarean section, and at risk of inducing subsequent type 2 diabetes mellitus (T2DM) and cardiovascular diseases [6–11]. Infants born from GDM women could be prone to abnormal fetal development such as being in macrosomia, having more congenital abnormalities, and having neonatal hypoglycemia [6, 12, 13]. Consequently, it is suggested that healthcare policy makers should be aware of the significance of GDM for early detection and further intervention.

To date, various relevant factors have been identified as predictors of GDM. Several studies have demonstrated that the frequently reported risk factors of GDM include older maternal ages, prepregnancy obesity, family history of diabetes (FHD) [14, 15], previous obstetric outcomes (e.g., macrosomia [16], stillbirth [17], abortion [18], premature delivery [19], congenital anomaly [16], being primigravida [20]), history of GDM [21], infection factors (e.g., Human Immunodeficiency Virus (HIV) [22]), pregestational smoking [23], and socioeconomic factors (educational level, occupation, and monthly household income) [24]. However, there are other evidences suggesting that maternal age, FHD, prepregnancy overweight or obesity, previous history of abortion, stillbirth, and macrosomia showed no significant association with GDM [25, 26]. Since most of the information regarding the main factors involved in GDM lack comprehensive analysis, it is necessary to conduct a meta-analysis to further explore the potential factors responsible for GDM.

## 2. Materials and Methods

Our study has been approved by the Open Science Framework (OSF) registries (https://osf.io/registries), and the registration number is 10.17605/OSF.IO/4HJGN. This meta-analysis was performed according to the Preferred Reporting Items for Systematic Review and Meta-Analysis (PRISMA) statement. Since this study was based on a meta-analysis of published studies, it did not require patient consent and ethical approval.

### 2.1. Literature Search Strategy

Four online databases (Web of Science, Embase, PubMed, and Cochrane Library) were systematically searched for articles published till August 20, 2020. We searched PubMed using the following terms: “diabetes mellitus” OR “diabetes” AND “pregnancy” OR “pregnancies” OR “gestation” OR “diabetes, gestational” OR “diabetes, pregnancy-induced” OR “diabetes, pregnancy induced” OR “pregnancy induced diabetes” OR “gestational diabetes” OR “diabetes mellitus gestational” OR “gestational diabetes mellitus” AND “risk factor” OR “risk factors.”

### 2.2. Inclusion and Exclusion Criteria

Inclusion criteria include the following: (1) women with GDM (the observation group) and with healthy pregnancies (the control group); (2) the reported relevant factors in our studies including maternal age ≥ 25 years, prepregnancy overweight or obese, history of GDM, primigravida, history of congenital anomaly, FHD, history of macrosomia, HIV status, history of stillbirth, history of premature delivery, history of abortion, and pregestational smoking; and (3) observational studies.

Exclusion criteria include the following: (1) studies not published in English; (2) meta-analyses, reviews, conference summaries, case reports, letters, and guidelines; and (3) animal experiments.

### 2.3. Data Extraction and Quality Assessment

The data were extracted by two reviewers (Yu Zhang and Cheng-Ming Xiao) independently according to the inclusion and exclusion criteria. If a conflict existed, the third reviewer (Yi-Meng Gao) would join in extracting the data. The following study features were extracted from each article: the first author's name, year of publication, country, study design, maternal age (years), sample size, the number of GDM cases, and quality assessment scores. The revised Joanna Briggs Institute (JBI) scale was used for cross-sectional studies to evaluate the quality of the literature, with 1-13 being low-risk of bias, and 14-20 being high-risk of bias. The modified Newcastle-Ottawa Scale (NOS) was used for case-control studies and cohort studies, and the studies with scores of 1-4 were considered low quality, while those with scores of 5-10 were considered high quality.

### 2.4. Statistical Analysis

Data were analyzed using Stata 15.1 software (Stata Corporation, College Station, TX, USA). The factors were assessed by odds ratios (ORs) and 95% confidence intervals (CIs). Heterogeneity tests were performed for each effect size, and random-effects models were adopted when *I*^2^ ≥ 50%; otherwise, fixed effects models were performed. The publication bias was estimated using Egger's test and adjusted by trim and fill method. A difference was considered statistically significant at *P* < 0.05.

## 3. Results

### 3.1. Literature Search

**Figure 1:**
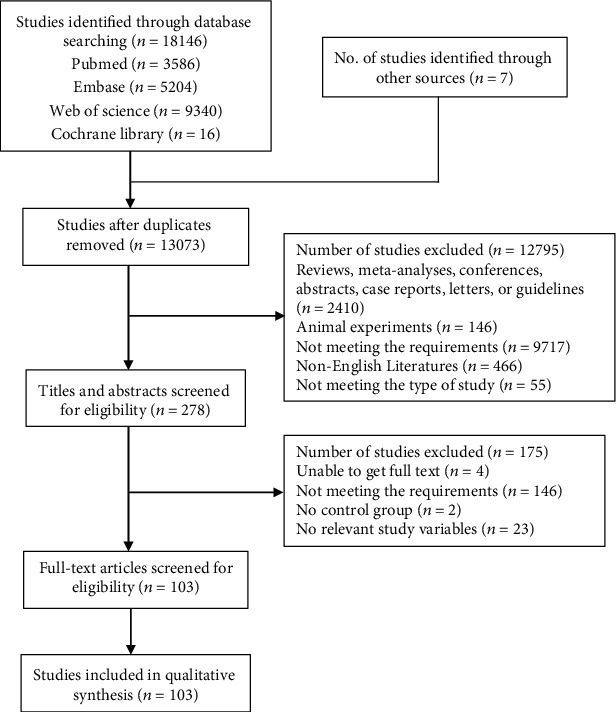
Flow diagram of search strategy.

In this study, 3,586 articles were extracted from PubMed, 5,204 from Embase, 9,340 from Web of Science, 16 from the Cochrane Central, and 7 from other sources. After the removal of duplicate records (*n* = 13,073), 278 articles were excluded after screening of the titles and abstracts and another 103 through full-text screening for eligibility. Finally, a total of 103 studies (Supplementary Material 1) were included in our study for evaluating the relationship between these factors and GDM. The flow diagram is shown in Figure 1.

### 3.2. Study Characteristics

A total of 1,826,454 pregnant women were enrolled in this meta-analysis, divided into the observation group (with GDM) composed of 120,696 subjects and the control group (without GDM) composed of 1,705,758 subjects. In terms of the quality of our included studies, scores from the assessment by the revised NOS and JBI scales were summarized in Table 1. The quality scores ranged from 4 to 16. Of the 103 included studies, 29 articles were low quality, while 74 were high quality (Table 1).

The numbers of the included studies according to different factors are as follows: maternal age (years) ≥ 25, *n* = 36; prepregnancy overweight or obese, *n* = 48; history of GDM, *n* = 24; primigravida, *n* = 56; history of congenital anomaly, *n* = 3; FHD, *n* = 74; history of macrosomia, *n* = 26; HIV status, *n* = 4; history of stillbirth, *n* = 11; history of abortion, *n* = 19; history of premature delivery, *n* = 3; and pregestational smoking, *n* = 9.

### 3.3. Factors Associated with GDM

**Figure 2:**
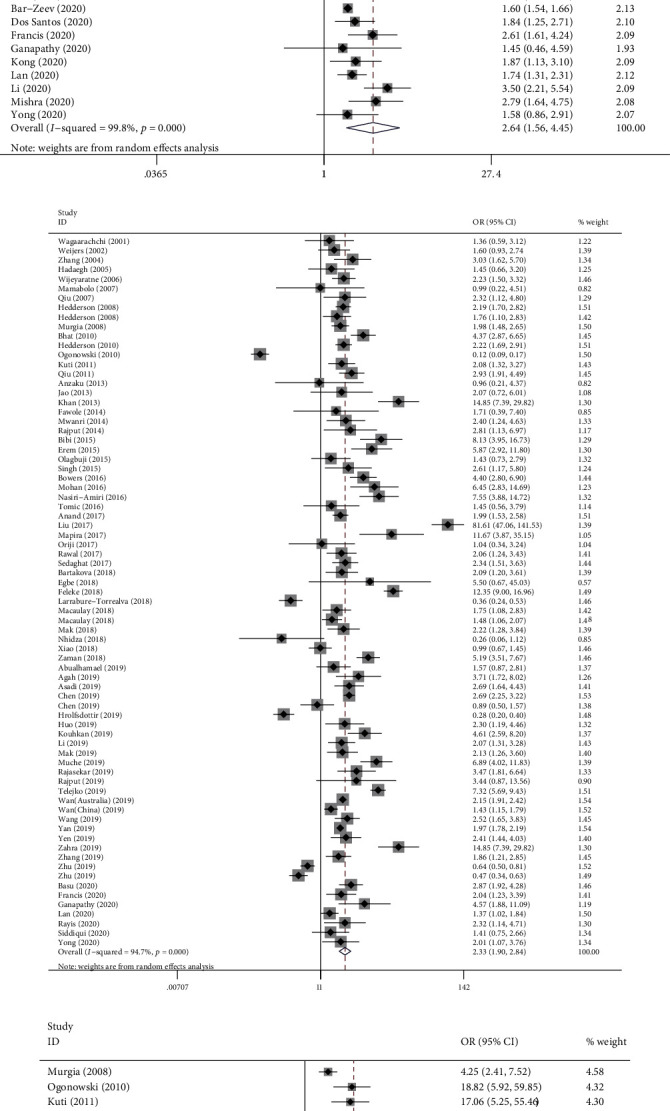
Forest plot for factors associated with GDM: (a) maternal age ≥ 25 years; (b) prepregnancy overweight or obese; (c) FHD; (d) history of GDM; (e) HIV status; (f) pregestational smoking.

**Figure 3:**
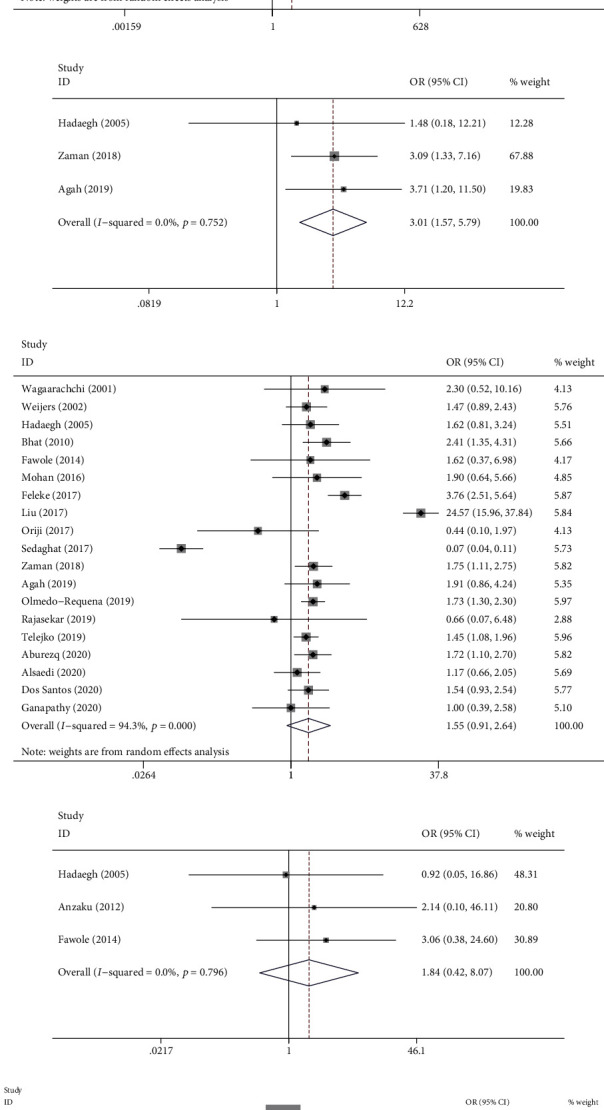
Forest plot for previous history of obstetric factors associated with GDM: (a) macrosomia; (b) stillbirth; (c) premature delivery; (d) abortion; (e) congenital anomaly; (f) primigravida.

The results demonstrated that maternal age ≥ 25 years (OR: 2.466, 95% CI: (2.121, 2.866), *P* < 0.001), prepregnancy overweight or obese (OR: 2.637, 95% CI: (1.561, 4.453), *P* < 0.001), history of GDM (OR: 21.137, 95% CI: (8.785, 50.858), *P* < 0.001), FHD (OR: 2.326, 95% CI: (1.904, 2.843), *P* < 0.001), history of macrosomia (OR: 2.539, 95% CI: (1.612, 4.000), *P* < 0.001), history of stillbirth (OR: 2.341, 95% CI: (1.435, 3.819), *P* = 0.001), history of premature delivery (OR: 3.013, 95% CI: (1.569, 5.787), *P* = 0.001), and pregestational smoking (OR: 2.322, 95% CI: (1.359, 3.967), *P* = 0.002) were associated with a higher risk of GDM. Nonetheless, there were no significant differences in terms of the history of congenital anomaly (OR: 1.837, 95% CI: (0.418, 8.067), *P* = 0.421), HIV status (OR: 1.168, 95% CI: (0.902, 1.512), *P* = 0.238), and history of abortion (OR: 1.546, 95% CI: (0.906, 2.639), *P* = 0.110). In addition, being primigravida (OR: 0.752, 95% CI: (0.698, 0.810), *P* < 0.001) was associated with the reduced risk of GDM (Table 2, Figures 2(a)–2(f) and 3(a)–3(f)).

### 3.4. Sensitivity Analysis and Publication Bias

**Figure 4:**
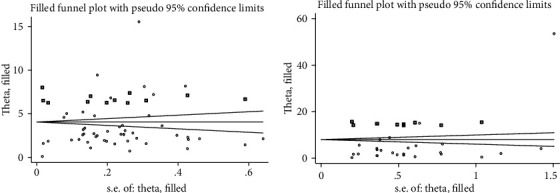
Egger's funnel plot of the publication bias improved by the trim and fill method for factors of GDM: (a) prepregnancy overweight or obese and (b) history of macrosomia.

Sensitivity analysis of each factor was conducted, and the results were found to have stability without any difference in homogeneity and the synthesized results, despite the change of the factors that affected the results (Supplementary Material 2). Results of Egger's test indicated that there was no significant publication bias in maternal age ≥ 25 (*t* = 0.19, *P* = 0.243), history of GDM (*t* = 1.83, *P* = 0.081), primigravida (*t* = −1.53, *P* = 0.132), FHD (*t* = 1.35, *P* = 0.181), history of stillbirth (*t* = −0.18, *P* = 0.862), and history of abortion (*t* = −0.26, *P* = 0.80). Prepregnancy overweight or obese (*t* = 4.85, *P* < 0.001) and history of macrosomia (*t* = 2.24, *P* = 0.035) showed a publication bias, and after adjustments by the trim and fill method, there was no obvious asymmetry in the funnel plots, meaning no publication bias was detected (Table 2, Figures 4(a)–4(b)).

## 4. Discussion

In this meta-analysis of 1,826,454 pregnant women from diverse international cohorts, our findings suggested that factors such as maternal age ≥ 25 years, prepregnancy overweight or obese, pregestational smoking, FHD, previous history of GDM, macrosomia, stillbirth, and premature delivery significantly increased the risk of GDM. Besides, being primigravida was associated with a lower risk of GDM, whereas history of congenital anomaly, HIV status, and history of abortion showed no impact on the risk of GDM; controlling these relevant factors for GDM could reduce the serious increase of the occurrence of GDM.

Maternal age was reported to be closely associated with GDM. Older maternal age increased the risk of developing GDM, and the threshold for lower risks was recommended as 25 years old by the American Diabetic Association [27], similar to the result of our meta-analysis. However, other studies differed with the result mentioned above, i.e., they recommended that maternal age greater than 35 years was more prone to GDM [20, 28]. Although it is shown that there is a certain difference in the cutoff value of maternal age, there is an inevitable risk of developing GDM with the annual increase of age in modern society [29]. The reason for increasing older ages at pregnancy may be related to the implementation of the universal two-child policy, especially in China, as well as a longer period of education and better access to birth control technologies.

Prepregnancy overweight or obese was another major risk factor identified in the current study. A study conducted by Mohan and Chandrakumar also demonstrated that prepregnancy weight management could reduce a woman's risk of GDM [30]. There were other studies with similar results to ours [31, 32], despite their varieties of dietary habits and with most people consuming large amounts of alcoholic beverages. Counselling for pregnant women should emphasize the need for women to avoid sedentary lifestyles before pregnancy and to be aware of the risks of GDM to both themselves and the unborn child.

Our study also suggested that FHD (particularly in a first-degree relative) was strongly related to an increased risk of GDM, which had been observed in a previous study [33]. This was partly because of an increased susceptibility to GDM due to a genetic deficiency in insulin secretion from their first-degree relatives [34]. Therefore, it is important to emphasize that healthcare education providers must obtain accurate personal or family history from their recipients in order to identify at-risk mothers for preventing GDM.

Another significant medical factor associated with a higher risk for developing GDM was history of GDM. Interestingly, a retrospective study [35] and two case-control studies [21, 36] also had similar results showing that history of GDM was thought to be a common risk factor in repeated pregnancies [34].

Among the obstetric factors of GDM, Anzaku and Musa pointed out that women with previous history of macrosomia were the only independent risk factor for GDM in the next pregnancy [16], which was similar to our results. A case-control study indicated that women having a history of abortion increased the risk of developing GDM at the central hospitals of the Amhara region, Ethiopia [34]. In contrast to this finding, our study showed no significant association between GDM and previous history of abortion, while another study showed a similar result to ours [19]. Limited literatures reported an association between a history of fetal congenital anomaly or premature delivery and GDM. Our results, supported by a previous study, revealed that they had no link [37]. However, women who had a history of premature delivery would be prone to the development of GDM, and it can be attributed to the intrauterine damage of the mother and the fetus [38]; however, more research is required to affirm this result. The current study also indicated that pregnant women with a history of stillbirth would have a higher risk of developing GDM during future pregnancies. This finding was in line with a review conducted in Africa by Muche et al. [23]. A study conducted in Pakistan demonstrated that the incidence of GDM in primigravida Pakistani women was <1% [39]. A previous meta-analysis of 5 included studies implied that being primigravida would reduce the risk of GDM [37]. Our study of a larger trial containing 56 relevant studies has reached the same conclusion.

As for infection factors, Egbe et al. found that there were 13 out of 200 (6.5%) HIV-positive respondents through analysis, but no association between HIV and GDM was observed [17]. This finding was consistent with our meta-analysis, which was also supported by the research of Jao et al. [22] and a previous meta-analysis study conducted by Natamba et al. [37]. Because few studies have reported a link between HIV and GDM, their association still needs to be further explored through more researches.

With the exception of the most common risk factors, such as maternal age, prepregnancy overweight or obese, FHD, obstetric factors, and infection factors, this study demonstrated that there was a significant correlation between pregestational smoking and GDM. Previous studies have also noted that pregestational smoking was considered to be a risk factor, although its association has been rarely investigated at present [40]. This condition might be explained by the fact that there were several limitations in the way data collection related to smoking was conducted in our study. A recent systematic review examined the relationship between pregestational smoking and the risk of GDM, but no correlation was found [36]. The aspect of smoking in the development of GDM deserves further investigations. Possible uncontrolled confounding factors should be considered, such as the differences in socioeconomic status between groups, selection bias, or even passive smoking.

Strengths and limitations should be taken into account in further interpreting our findings. In terms of strengths, due to the high prevalence of GDM, our meta-analysis included studies conducted in different countries such as China, USA, Australia, and India, covering a number of nationwide representative populations, which to a certain extent had reduced the possible selection bias and reaching some relatively generalized conclusions. Nevertheless, the present study also had some limitations. Firstly, the role of confounding factors cannot be completely eliminated in our observational studies. Although majority of the articles included in the analysis evaluated multiple factors, limited studies have shown the association between other variables such as living quarters, substance abuse, dietary diversity, and physical activity issues with GDM. Prospective review studies need to clarify the correlation between GDM and the other factors mentioned above. Secondly, there is a high heterogeneity in our results which might also be attributed to the different demographic characteristics among populations in more than 37 countries covered by this meta-analysis. Additionally, qualitative studies about the reasons for GDM pathologically should be added in this review.

## 5. Conclusions

In our study, maternal age ≥ 25 years, prepregnancy overweight or obese, FHD, previous history of GDM, macrosomia, stillbirth and premature delivery, pregestational smoking, and being primigravida were considered as all independent risk factors of GDM. It is strongly recommended that all pregnant women in the future be screened early for GDM, especially those identified at higher risks of GDM, thereby leading to early diagnosis of GDM and early intervention.

## Figures and Tables

**Table 1:** Baseline characteristics of the included studies.

Author	Year	Country	Study design	Maternal age (years)	Sample sizes	GDM cases	Quality scores
Wagaarachchi	2001	Sri Lanka	Case-control	—	1004	41	5
Weijers	2002	Amsterdam	Case-control	25.2 ± 4.5	561	71	5
Yang	2002	China	Case-control	28.0 ± 0.28	9886	177	4
Dempsey	2004	USA	Case-control	—	541	155	6
Ozumba	2004	Nigeria	Case-control	—	400	200	5
Zhang	2004	China	Case-control	—	327	67	6
Hadaegh	2005	Iran	Case-control	—	700	62	6
Janghorbani	2006	UK	Case-control	—	3933	65	4
Wijeyaratne	2006	Sri Lanka	Case-control	—	442	274	5
Mamabolo	2007	South Africa	Case-control	29.0 ± 8.5	262	23	4
Qiu	2007	USA	Case-control	33.1 ± 0.6	201	105	5
Cypryk	2008	Poland	Case-control	—	1670	510	4
Hedderson	2008	USA	Case-control	—	1323	381	6
Hedderson	2008	USA	Case-control	—	455	251	6
Murgia	2008	Italy	Case-control	32.8 ± 0.2	1103	247	5
Bhat	2010	India	Case-control	26.63 ± 4.547	600	300	4
Harizopoulou	2010	Greece	Cross-sectional	33.8 ± 4.5	160	40	5
Hedderson	2010	USA	Case-control	—	1134	341	5
Ogonowski	2010	Poland	Case-control	30.2 ± 5.6	2425	1414	6
Kuti	2011	Nigeria	Case-control	—	765	106	4
Morisset	2011	Canada	Case-control	31.5 ± 5.1	294	55	5
Qiu	2011	USA	Case-control	32.9 ± 5.3	596	185	5
Anzaku	2013	Nigeria	Cross-sectional	31.2 ± 5.8	253	21	5
Jao	2013	Cameroon	Cross-sectional	30.5 (27.5-34.5)	316	20	4
Khan	2013	Pakistan	Case-control	35.01 ± 4.54	200	103	5
Fawole	2014	Ibadan	Cross-sectional	—	1086	35	12
Kirke	2014	Australia	Case-control	30.8 ± 5.7	1636	73	4
Mwanri	2014	Tanzania	Cross-sectional	—	910	54	14
Padmanabhan	2014	Australia	Case-control	33.0 (29.0-36.0)	682	343	4
Rajput	2014	India	Case-control	24.0 ± 3.1	913	127	6
Tabatabaei	2014	Canada	Case-control	30.8 ± 0.7	96	48	4
Bibi	2015	Pakistan	Cross-sectional	—	190	50	11
Erem	2015	Turkey	Cross-sectional	32.4 ± 3.9	815	39	15
Olagbuji	2015	Nigeria	Cohort	—	1059	91	5
Oppong	2015	Ghana	Cross-sectional	—	399	37	14
Robledo	2015	USA	Cohort	—	649952	11334	5
Singh	2015	India	Case-control	29.05 ± 3.55	102	51	5
Bowers	2016	Danish	Case-control	32.2 ± 4.3	699	350	4
Mohan	2016	India	Case-control	—	201	32	4
Nasiri-Amiri	2016	Iran	Case-control	—	200	100	6
Tomic	2016	Bosnia and Herzegovina	Cross-sectional	—	285	31	13
Abdelmola	2017	Saudi Arabia	Cross-sectional	—	36	36	14
Anand	2017	Canada	Case-control	31.2 ± 4.0	1006	365	6
Collier	2017	UK	Case-control	—	47290	973	4
Farina	2017	Italy	Case-control	33.5 (24-40)	72	12	6
Liu	2017	China	Case-control	29 ± 5.2	600	300	6
Mapira	2017	Rwanda	Cross-sectional	—	288	24	5
Oriji	2017	Nigeria	Case-control	—	235	35	5
Rawal	2017	USA	Case-control	30.5 ± 5.7	321	107	5
Sedaghat	2017	Iran	Case-control	29.64 ± 4.52	388	122	6
Sugiyama	2017	Palau	Case-control	—	1730	95	5
Bartakova	2018	Czech	Case-control	33 (29-36)	363	293	4
Egbe	2018	Cameroon	Cross-sectional	—	200	41	13
Feleke	2018	Ethiopia	Case-control	—	2257	567	5
Larrabure-Torrealva	2018	America	Cross-sectional	29.83 ± 6.49	1300	205	15
Macaulay	2018	South Africa	Cohort	31 (27-36)	741	83	7
Macaulay	2018	South Africa	Cross-sectional	31 (27-36)	1900	174	15
Mak	2018	China	Cohort	26.8 ± 4.2	1337	199	6
Nhidza	2018	Zimbabwe	Cross-sectional	—	150	10	5
Wu	2018	China	Case-control	32.0 ± 4.32	4959	1080	6
Xiao	2018	China	Case-control	32 (29-34)	1585	599	5
Zaman	2018	Iran	Cross-sectional	29.72 ± 5.34	520	260	16
Abualhamael	2019	Saudi Arabia	Case-control	33.4 ± 5.9	196	103	7
Agah	2019	Iran	Cross-sectional	—	609	28	14
Asadi	2019	Iran	Case-control	29.00 ± 5.17	278	130	6
Chakkalakal	2019	Tennessee	Case-control	29.27 ± 5.14	89	40	4
Chen	2019	China	Case-control	—	9556	1464	4
Chen	2019	China	Case-control	31.28 ± 4.66	249	123	5
Hrolfsdottir	2019	Iceland	Cohort	31.8 ± 5.4	1651	264	6
Hu	2019	China	Cohort	—	1014	238	5
Huo	2019	China	Case-control	29.2 ± 2.7	486	243	7
Ijas	2019	Finland	Cohort	—	24577	5680	5
Kouhkan	2019	Iran	Case-control	32.15 ± 5.07	270	135	6
Li	2019	China	Case-control	30.03 ± 3.73	496	248	4
Mak	2019	China	Cohort	27.4 ± 4.3	1449	229	6
Muche	2019	Ethiopia	Cross-sectional	—	1027	131	12
Olmedo-Requena	2019	Spain	Cross-sectional	33.5 ± 5.5	1466	291	16
Rajasekar	2019	Vellore	Cross-sectional	253.27 ± 4.42	225	75	16
Rajput	2019	India	Case-control	25.94 ± 4.90	100	50	7
Telejko	2019	Poland	Cohort	31 (27-35)	1508	397	7
Wan (China)	2019	China	Case-control	32.7 ± 4.9	3419	398	5
Wan (Australia)	2019	Australia	Case-control	31.9 ± 5.6	28594	1181	5
Wang	2019	China	Case-control	31.00 ± 4.53	1552	776	7
Yan	2019	China	Cohort	30.1 ± 4.5	78572	13846	7
Yen	2019	China	Cohort	—	527	74	5
Zahra	2019	Pakistan	Case-control	—	200	103	5
Zhang	2019	China	Cohort	29.0 (27-32)	2093	241	5
Zhu	2019	China	Case-control	28.1 ± 4.4	3110	399	5
Zhu	2019	China	Case-control	27.9 ± 4.3	3289	429	5
Aburezq	2020	Kuwait	Cross-sectional	31.45 ± 5.7	653	92	15
Alsaedi	2020	Saudi Arabia	Case-control	31.7 ± 6.6	347	279	5
Bar-Zeev	2020	Ohio	Case-control	—	222408	12897	5
Basu	2020	India	Case-control	25.78 ± 4.89	715	127	6
Dos Santos	2020	Brazil	Cross-sectional	—	2284	126	14
Francis	2020	USA	Case-control	30.5 ± 5.7	321	107	7
Ganapathy	2020	India	Case-control	29.54 ± 4.3	140	70	6
Giles	2020	Australia	Cross-sectional	—	671227	54805	12
Kong	2020	China	Cohort	27.9 ± 3.1	1441	114	6
Lan	2020	China	Cohort	29.6 ± 4.2	1910	620	6
Li	2020	China	Case-control	30.6 ± 4.4	610	305	5
Mishra	2020	India	Case-control	—	373	100	5
Rayis	2020	Saudi Arabia	Case-control	30 (25-34)	259	48	4
Siddiqui	2020	Saudi Arabia	Cross-sectional	32.9 ± 5.5	218	53	16
Yong	2020	The Netherlands	Cohort	29.80 ± 4.39	452	48	5

GDM: gestational diabetes mellitus.

**Table 2:** Summary of the meta-analysis of associated factors for GDM.

No.	Factors	No. studies included	OR	95% CI	*I* ^2^	*P* heterogeneity	*t*	Bias *P* heterogeneity
1	Maternal age ≥ 25 years	36	2.466	2.121, 2.866	96.2	<0.001	0.19	0.243
2	Prepregnancy overweight or obese	48	2.637	1.561, 4.453	99.8	<0.001	4.85	0.001
3	FHD	74	2.326	1.904, 2.843	94.7	<0.001	1.83	0.081
4	Primigravida	56	0.752	0.698, 0.810	94.7	<0.001	1.53	0.132
5	History of congenital anomaly	3	1.837	0.418, 8.067	0.0	0.421	—	—
6	History of GDM	24	21.137	8.785, 50.858	96.9	<0.001	1.35	0.181
7	History of macrosomia	26	2.539	1.612, 4.000	86.6	<0.001	2.24	0.035
8	HIV status	4	1.168	0.902, 1.512	0.0	0.238	—	—
9	History of stillbirth	11	2.341	1.435, 3.819	52.0	0.001	0.18	0.862
10	History of abortion	19	1.546	0.906, 2.639	94.3	0.110	0.26	0.800
11	History of premature delivery	3	3.013	1.569, 5.787	0.0	0.001	—	—
12	Pregestational smoking	9	2.322	1.359, 3.967	66.7	0.002	—	—

CI: confidence interval; FHD: family history of diabetes mellitus; GDM: gestational diabetes mellitus; HIV: human immunodeficiency virus; OR: odds ratio.

## Data Availability

The data used to support the findings of this study are included within the article.

## Supplementary Materials

Supplementary Materials References of included studies. Sensitivity analysis: (A) maternal age ≥ 25 years; (B) prepregnancy overweight or obese; (C) FHD; (D) history of GDM; (E) HIV status; (F) pregestational smoking; (G) history of macrosomia; (H) history of stillbirth; (I) history of premature delivery; (J) history of abortion; (K) history of congenital anomaly; (L) primigravida.
