# Supplementary material for: Factors Associated with Gestational Diabetes Mellitus: A Meta-Analysis
Source: J Diabetes Res. 2021 May 10;2021:6692695. doi: 10.1155/2021/6692695 (PMC8128547; doi:10.1155/2021/6692695)
Supplement: Supplementary Materials — References of included studies. Sensitivity analysis: (A) maternal age ≥ 25 years; (B) prepregnancy overweight or obese; (C) FHD; (D) history of GDM; (E) HIV status; (F) pregestational smoking; (G) history of macrosomia; (H) history of stillbirth; (I) history of premature delivery; (J) history of abortion; (K) history of congenital anomaly; (L) primigravida. [file 6692695.f1.zip › 6692695.f1.docx]

**References:**

[1-103]

1. Wagaarachchi PT, Fernando L, Premachadra P, Fernando DJS: **Screening based on risk factors for gestational diabetes in an Asian population**. *Journal of Obstetrics and Gynaecology* 2001, **21**(1):32-34.

2. Weijers RNM, Bekedam DJ, Smulders YM: **Determinants of mild gestational hyperglycemia and gestational diabetes mellitus in a large Dutch multiethnic cohort**. *Diabetes Care* 2002, **25**(1):72-77.

3. Yang X, Hsu-Hage B, Zuang H, Yu L, Dong L, Li J, Shao P, Zhang C: **Gestational diabetes mellitus in women of single gravidity in Tianjin City, China**. *Diabetes Care* 2002, **25**(5):847-851.

4. Dempsey JC, Butler CL, Sorensen TK, Lee IM, Thompson ML, Miller RS, Frederick IO, Williams MA: **A case-control study of maternal recreational physical activity and risk of gestational diabetes mellitus**. *Diabetes Research and Clinical Practice* 2004, **66**(2):203-215.

5. Ozumba BC, Obi SN, Oli JM: **Diabetes mellitus in pregnancy in an African population**. *International journal of gynaecology and obstetrics: the official organ of the International Federation of Gynaecology and Obstetrics* 2004, **84**(2):114-119.

6. Zhang CL, Williams MA, Frederick IO, King IB, Sorensen TK, Kestin MM, Dashow EE, Luthy DA: **Vitamin C and the risk of gestational diabetes mellitus - A case-control study**. *Journal of Reproductive Medicine* 2004, **49**(4):257-266.

7. Hadaegh F, Tohidi M, Harati H, Kheirandish M, Rahimi S: **Prevalence of gestational diabetes mellitus in southern Iran (Bandar Abbas City)**. *Endocrine practice : official journal of the American College of Endocrinology and the American Association of Clinical Endocrinologists* 2005, **11**(5):313-318.

8. Janghorbani M, Stenhouse EA, Jones RB, Millward BA: **Is neighbourhood deprivation a risk factor for gestational diabetes mellitus?** *Diabetic Medicine* 2006, **23**(3):313-317.

9. Wijeyaratne CN, Waduge R, Arandara D, Arasalingam A, Sivasuriam A, Dodampahala SH, Balen AH: **Metabolic and polycystic ovary syndromes in indigenous South Asian women with previous gestational diabetes mellitus**. *Bjog-an International Journal of Obstetrics and Gynaecology* 2006, **113**(10):1182-1187.

10. Mamabolo RL, Alberts M, Levitt NS, Delemarre-van de Waal HA, Steyn NP: **Prevalence of gestational diabetes mellitus and the effect of weight on measures of insulin secretion and insulin resistance in third-trimester pregnant rural women residing in the Central Region of Limpopo Province, South Africa**. *Diabetic Medicine* 2007, **24**(3):233-239.

11. Qiu C, Rudra C, Austin MA, Williams MA: **Association of gestational diabetes mellitus and low-density lipoprotein (LDL) particle size**. *Physiological Research* 2007, **56**(5):571-578.

12. Cypryk K, Szymczak W, Czupryniak L, Sobczak M, Lewiński A: **Gestational diabetes mellitus - An analysis of risk factors**. *Endokrynologia Polska* 2008, **59**(5):393-397.

13. Hedderson MM, Ferrara A: **High Blood Pressure Before and During Early Pregnancy Is Associated With an Increased Risk of Gestational Diabetes Mellitus**. *Diabetes Care* 2008, **31**(12):2362-2367.

14. Hedderson MM, Williams MA, Holt VL, Weiss NS, Ferrara A: **Body mass index and weight gain prior to pregnancy and risk of gestational diabetes mellitus**. *American Journal of Obstetrics and Gynecology* 2008, **198**(4).

15. Murgia C, Orru M, Portoghese E, Garau N, Zedda P, Berria R, Motzo C, Sulis S, Murenu M, Paoletti AM *et al*: **Autoimmunity in gestational diabetes mellitus in Sardinia: a preliminary case-control report**. *Reproductive Biology and Endocrinology* 2008, **6**.

16. Bhat M, K NR, Sarma SP, Menon S, C VS, S GK: **Determinants of gestational diabetes mellitus: A case control study in a district tertiary care hospital in south India**. *Int J Diabetes Dev Ctries* 2010, **30**(2):91-96.

17. Harizopoulou VC, Kritikos A, Papanikolaou Z, Saranti E, Vavilis D, Klonos E, Papadimas I, Goulis DG: **Maternal physical activity before and during early pregnancy as a risk factor for gestational diabetes mellitus**. *Acta Diabetologica* 2010, **47**:S83-S89.

18. Hedderson MM, Gunderson EP, Ferrara A: **Gestational Weight Gain and Risk of Gestational Diabetes Mellitus**. *Obstetrics and Gynecology* 2010, **115**(3):597-604.

19. Ogonowski J, Miazgowski T: **Are short women at risk for gestational diabetes mellitus?** *European Journal of Endocrinology* 2010, **162**(3):491-497.

20. Kuti MA, Abbiyesuku FM, Akinlade KS, Akinosun OM, Adedapo KS, Adeleye JO, Adesina OA: **Oral glucose tolerance testing outcomes among women at high risk for gestational diabetes mellitus**. *Journal of Clinical Pathology* 2011, **64**(8):718-721.

21. Morisset AS, Tchernof A, Dubé MC, Veillette J, Weisnagel SJ, Robitaille J: **Weight gain measures in women with gestational diabetes mellitus**. *Journal of Women's Health* 2011, **20**(3):375-380.

22. Qiu C, Frederick IO, Zhang C, Sorensen TK, Enquobahrie DA, Williams MA: **Risk of Gestational Diabetes Mellitus in Relation to Maternal Egg and Cholesterol Intake**. *American Journal of Epidemiology* 2011, **173**(6):649-658.

23. Anzaku AS, Musa J: **Prevalence and associated risk factors for gestational diabetes in Jos, North-central, Nigeria**. *Archives of Gynecology and Obstetrics* 2013, **287**(5):859-863.

24. Jao J, Wong M, Van Dyke RB, Geffner M, Nshom E, Palmer D, Muffih PT, Abrams EJ, Sperling RS, Leroith D: **Gestational diabetes mellitus in HIV-infected and -uninfected pregnant women in Cameroon**. *Diabetes Care* 2013, **36**(9):e141-142.

25. Khan R, Ali K, Khan Z: **Socio-demographic Risk Factors of Gestational Diabetes Mellitus**. *Pak J Med Sci* 2013, **29**(3):843-846.

26. Fawole AO, Ezeasor C, Bello FA, Roberts A, Awoyinka BS, Tongo O, Adeleye JO, Ipadeola A: **Effectiveness of a structured checklist of risk factors in identifying pregnant women at risk of gestational diabetes mellitus: A cross-sectional study**. *Nigerian Journal of Clinical Practice* 2014, **17**(4):495-501.

27. Kirke AB, Evans SF, Walters BN: **Gestational diabetes in a rural, regional centre in south Western Australia: predictors of risk**. *Rural and remote health* 2014, **14**(3):2667.

28. Mwanri AW, Kinabo J, Ramaiya K, Feskens EJ: **Prevalence of gestational diabetes mellitus in urban and rural Tanzania**. *Diabetes Res Clin Pract* 2014, **103**(1):71-78.

29. Padmanabhan S, Wagstaff A, Tung V, Chan YF, Bartlett A, Lau SM: **Increase in body mass index during pregnancy and risk of gestational diabetes**. *Diabetes Research and Clinical Practice* 2014, **106**(3):E79-E82.

30. Rajput M, Bairwa M, Rajput R: **Prevalence of gestational diabetes mellitus in rural Haryana: A community-based study**. *Indian Journal of Endocrinology and Metabolism* 2014, **18**(3):350-354.

31. Tabatabaei N, Giguère Y, Forest JC, Rodd CJ, Kremer R, Weiler HA: **Osteocalcin is higher across pregnancy in Caucasian women with gestational diabetes mellitus**. *Can J Diabetes* 2014, **38**(5):307-313.

32. Bibi S, Saleem U, Mahsood N: **The frequency of gestational diabetes mellitus and associated risk factors at khyber teaching hospital Peshawar**. *Journal of Postgraduate Medical Institute* 2015, **29**(1):43-46.

33. Erem C, Kuzu UB, Deger O, Can G: **Prevalence of gestational diabetes mellitus and associated risk factors in Turkish women: The Trabzon GDM Study**. *Archives of Medical Science* 2015, **11**(4):724-735.

34. Olagbuji BN, Atiba AS, Olofinbiyi BA, Akintayo AA, Awoleke JO, Ade-Ojo IP, Fasubaa OB: **Prevalence of and risk factors for gestational diabetes using 1999, 2013 WHO and IADPSG criteria upon implementation of a universal one-step screening and diagnostic strategy in a sub-Saharan African population**. *European Journal of Obstetrics and Gynecology and Reproductive Biology* 2015, **189**:27-32.

35. Oppong SA, Ntumy MY, Amoakoh-Coleman M, Ogum-Alangea D, Modey-Amoah E: **Gestational diabetes mellitus among women attending prenatal care at Korle-Bu Teaching Hospital, Accra, Ghana**. *International Journal of Gynecology & Obstetrics* 2015, **131**(3):246-250.

36. Robledo CA, Mendola P, Yeung E, Maennistoe T, Sundaram R, Liu D, Ying Q, Sherman S, Grantz KL: **Preconception and early pregnancy air pollution exposures and risk of gestational diabetes mellitus**. *Environmental Research* 2015, **137**:316-322.

37. Singh S, Urooj A: **Influence of Pre-Pregnancy Weight, Food Habits and Lifestyle on Gestational Diabetes**. *Current Research in Nutrition and Food Science* 2015, **3**(2):156-164.

38. Bowers KA, Olsen SF, Bao W, Halldorsson TI, Strøm M, Zhang C: **Plasma concentrations of ferritin in early pregnancy are associated with risk of gestational diabetes mellitus in women in the Danish National Birth Cohort**. *Journal of Nutrition* 2016, **146**(9):1756-1761.

39. Mohan MA, Chandrakumar A: **Evaluation of prevalence and risk factors of gestational diabetes in a tertiary care hospital in Kerala**. *Diabetes and Metabolic Syndrome: Clinical Research and Reviews* 2016, **10**(2):68-71.

40. Nasiri-Amiri F, Bakhtiari A, Faramarzi M, Rad HA, Pasha H: **The Association Between Physical Activity During Pregnancy and Gestational Diabetes Mellitus: A Case-Control Study**. *International Journal of Endocrinology and Metabolism* 2016, **14**(3).

41. Tomic V, Misic M, Simic AD, Boskovic A, Kresic T, Peric O, Orlovic M, Blagojevic IC: **PREVALENCE OF GESTATIONAL DIABETES IN THE SOUTHERN PART OF BOSNIA AND HERZEGOVINA**. *Materia socio-medica* 2016, **28**(5):329-332.

42. Abdelmola AO, Mahfouz MS, Gahtani MAM, Mouharrq YJ, Hakami BHO, Daak OI, Alharbi AQ, Ali Masmali UM, Melassy DAM, Alhazmi AA: **Gestational diabetes prevalence and risk factors among pregnant women — Jazan Region, Saudi Arabia**. *Clinical Diabetology* 2017, **6**(5):172-177.

43. Anand SS, Gupta M, Teo KK, Schulze KM, Desai D, Abdalla N, Zulyniak M, de Souza R, Wahi G, Shaikh M *et al*: **Causes and consequences of gestational diabetes in South Asians living in Canada: results from a prospective cohort study**. *CMAJ open* 2017, **5**(3):E604-e611.

44. Collier A, Abraham EC, Armstrong J, Godwin J, Monteath K, Lindsay R: **Reported prevalence of gestational diabetes in Scotland: The relationship with obesity, age, socioeconomic status, smoking and macrosomia, and how many are we missing?** *Journal of Diabetes Investigation* 2017, **8**(2):161-167.

45. Farina A, Eklund E, Bernabini D, Paladino M, Righetti F, Monti G, Lambert-Messerlian G: **A First-Trimester Biomarker Panel for Predicting the Development of Gestational Diabetes**. *Reproductive Sciences* 2017, **24**(6):954-959.

46. Liu L-Y, Zhang Y-L, Li L: **Risk factor of gestational diabetes among healthy Chinese women: an observational study**. *Biomedical Research-India* 2017, **28**(5):2126-2130.

47. Mapira HT, Tumusiime DK, Yarasheski K, Rujeni N, Cade TW, Mutimura E: **Strategy to improve the burden of gestational diabetes in African women: Rwandan perspective**. *Rwanda Journal* 2017, **4**(1):36.

48. Oriji VK, Ojule JD, Fumudoh BO: **Prediction of Gestational Diabetes Mellitus in Early Pregnancy: Is Abdominal Skin Fold Thickness 20 mm or More an Independent Risk Predictor?** *Journal of Biosciences and Medicines* 2017, **05**(11):13-26.

49. Rawal S, Hinkle SN, Bao W, Zhu Y, Grewal J, Albert PS, Weir NL, Tsai MY, Zhang C: **A longitudinal study of iron status during pregnancy and the risk of gestational diabetes: findings from a prospective, multiracial cohort**. *Diabetologia* 2017, **60**(2):249-257.

50. Sedaghat F, Akhoondan M, Ehteshami M, Aghamohammadi V, Ghanei N, Mirmiran P, Rashidkhani B: **Maternal Dietary Patterns and Gestational Diabetes Risk: A Case-Control Study**. *Journal of Diabetes Research* 2017, **2017**.

51. Sugiyama MS, Cash HL, Roseveare C, Reklai R, Basilius K, Madraisau S: **Assessment of Gestational Diabetes and Associated Risk Factors and Outcomes in the Pacific Island Nation of Palau**. *Maternal and child health journal* 2017, **21**(10):1961-1966.

52. Bartakova V, Kuricova K, Zlamal F, Belobradkova J, Kankova K: **Differences in food intake and genetic variability in taste receptors between Czech pregnant women with and without gestational diabetes mellitus**. *European Journal of Nutrition* 2018, **57**(2):513-521.

53. Egbe TO, Tsaku ES, Tchounzou R, Ngowe MN: **Prevalence and risk factors of gestational diabetes mellitus in a population of pregnant women attending three health facilities in Limbe, Cameroon: A cross-sectional study**. *Pan African Medical Journal* 2018, **31**.

54. Feleke BE: **Determinants of gestational diabetes mellitus: a case-control study**. *The journal of maternal-fetal & neonatal medicine : the official journal of the European Association of Perinatal Medicine, the Federation of Asia and Oceania Perinatal Societies, the International Society of Perinatal Obstet* 2018, **31**(19):2584-2589.

55. Larrabure-Torrealva GT, Martinez S, Luque-Fernandez MA, Sanchez SE, Mascaro PA, Ingar H, Castillo W, Zumaeta R, Grande M, Motta V *et al*: **Prevalence and risk factors of gestational diabetes mellitus: Findings from a universal screening feasibility program in Lima, Peru**. *BMC Pregnancy and Childbirth* 2018, **18**(1).

56. Macaulay S, Munthali RJ, Dunger DB, Norris SA: **The effects of gestational diabetes mellitus on fetal growth and neonatal birth measures in an African cohort**. *Diabet Med* 2018, **35**(10):1425-1433.

57. Macaulay S, Ngobeni M, Dunger DB, Norris SA: **The prevalence of gestational diabetes mellitus amongst black South African women is a public health concern**. *Diabetes Research and Clinical Practice* 2018, **139**:278-287.

58. Mak JKL, Ngoc Minh P, Lee AH, Tang L, Pan X-F, Binns CW, Sun X: **Dietary patterns during pregnancy and risk of gestational diabetes: a prospective cohort study in Western China**. *Nutrition Journal* 2018, **17**.

59. Nhidza G, Mutsaka K, Malunga G, Zhou DT: **Diagnosis of Gestational Diabetes Mellitus in Urban Harare, Zimbabwe**. *The Open Public Health Journal* 2018, **11**(1):1-7.

60. Wu L, Han L, Zhan Y, Cui L, Chen W, Ma L, Lv J, Pan R, Zhao D, Xiao Z: **Prevalence of gestational diabetes mellitus and associated risk factors in pregnant Chinese women: a cross-sectional study in Huangdao, Qingdao, China**. *Asia Pacific journal of clinical nutrition* 2018, **27**(2):383-388.

61. Xiao D, Chenhong W, Yanbin X, Lu Z: **Gestational diabetes mellitus and first trimester pregnancy-associated plasma protein A: A case–control study in a Chinese population**. *Journal of Diabetes Investigation* 2018, **9**(1):204-210.

62. Zaman F, Nouhjah S, Shahbazian H, Shahbazian N, Latifi SM, Jahanshahi A: **Risk factors of gestational diabetes mellitus using results of a prospective population-based study in Iranian pregnant women**. *Diabetes and Metabolic Syndrome: Clinical Research and Reviews* 2018, **12**(5):721-725.

63. Abualhamael S, Mosli H, Baig M, Noor AM, Alshehri FM: **Prevalence and associated risk factors of gestational diabetes mellitus at a university hospital in Saudi Arabia**. *Pakistan Journal of Medical Sciences* 2019, **35**(2):325-329.

64. Agah J, Roodsarabi F, Manzuri A, Amirpour M, Hosseinzadeh A: **Prevalence and associated risk factors of gestational diabetes mellitus in a tertiary hospital in Iran**. *Clinical and experimental obstetrics & gynecology* 2019, **46**(1):85-89.

65. Asadi M, Shahzeidi M, Nadjarzadeh A, Yusefabad HH, Mansoori A: **The relationship between pre-pregnancy dietary patterns adherence and risk of gestational diabetes mellitus in Iran: A case-control study**. *Nutrition & Dietetics* 2019, **76**(5):597-603.

66. Chakkalakal RJ, Hackstadt AJ, Trochez R, Gregory R, Elasy TA: **Gestational Diabetes and Maternal Weight Management During and After Pregnancy**. *Journal of Womens Health* 2019, **28**(5):646-653.

67. Chen Q, Feng Y, Yang H, Wu W, Zhang P, Wang K, Wang Y, Ko J, Shen J, Guo L *et al*: **A Vitamin Pattern Diet Is Associated with Decreased Risk of Gestational Diabetes Mellitus in Chinese Women: Results from a Case Control Study in Taiyuan, China**. *Journal of Diabetes Research* 2019, **2019**.

68. Chen X, Jiang X, Huang X, He H, Zheng J: **Association between Probiotic Yogurt Intake and Gestational Diabetes Mellitus: A Case-Control Study**. *Iranian journal of public health* 2019, **48**(7):1248-1256.

69. Hrolfsdottir L, Gunnarsdottir I, Birgisdottir BE, Hreidarsdottir IT, Smarason AK, Hardardottir H, Halldorsson TI: **Can a Simple Dietary Screening in Early Pregnancy Identify Dietary Habits Associated with Gestational Diabetes?** *Nutrients* 2019, **11**(8).

70. Hu J, Oken E, Aris IM, Lin P-ID, Ma Y, Ding N, Gao M, Wei X, Wen D: **Dietary Patterns during Pregnancy Are Associated with the Risk of Gestational Diabetes Mellitus: Evidence from a Chinese Prospective Birth Cohort Study**. *Nutrients* 2019, **11**(2).

71. Huo X, Li J, Cao Y-F, Li S-N, Shao P, Leng J, Li W, Liu J, Yang K, Ma RCW *et al*: **Trimethylamine N-Oxide Metabolites in Early Pregnancy and Risk of Gestational Diabetes: A Nested Case-Control Study**. *Journal of Clinical Endocrinology & Metabolism* 2019, **104**(11):5529-5539.

72. Ijas H, Koivunen S, Raudaskoski T, Kajantie E, Gissler M, Vaarasmaki M: **Independent and concomitant associations of gestational diabetes and maternal obesity to perinatal outcome: A register-based study**. *Plos One* 2019, **14**(8).

73. Kouhkan A, Khamseh ME, Moini A, Pirjani R, Arabipoor A, Zolfaghari Z, Hosseini R, Baradaran HR: **Diagnostic Accuracy of Body Mass Index and Fasting Glucose for The Prediction of Gestational Diabetes Mellitus after Assisted Reproductive Technology**. *International journal of fertility & sterility* 2019, **13**(1):32-37.

74. Li P, Yin J, Zhu Y, Li S, Chen S, Sun T, Shan Z, Wang J, Shang Q, Li X *et al*: **Association between plasma concentration of copper and gestational diabetes mellitus**. *Clinical Nutrition* 2019, **38**(6):2922-2927.

75. Mak JKL, Lee AH, Ngoc Minh P, Tang L, Pan X-F, Binns CW, Sun X: **Gestational diabetes and postnatal depressive symptoms: A prospective cohort study in Western China**. *Women and Birth* 2019, **32**(3):E427-E431.

76. Muche AA, Olayemi OO, Gete YK: **Prevalence of gestational diabetes mellitus and associated factors among women attending antenatal care at Gondar town public health facilities, Northwest Ethiopia**. *Bmc Pregnancy and Childbirth* 2019, **19**(1).

77. Olmedo-Requena R, Gomez-Fernandez J, Amezcua-Prieto C, Mozas-Moreno J, Khan KS, Jimenez-Moleon JJ: **Pre-Pregnancy Adherence to the Mediterranean Diet and Gestational Diabetes Mellitus: A Case-Control Study**. *Nutrients* 2019, **11**(5).

78. Rajasekar G, Muliyil DE, Cherian AG, Prasad JH, Mohan VR: **Prevalence and Factors Associated with Gestational Diabetes Mellitus among Antenatal Women at a Rural Health Center in Vellore**. *The Journal of the Association of Physicians of India* 2019, **67**(4):42-47.

79. Rajput R, Vohra S, Nanda S, Rajput M: **Severe 25(OH)vitamin-D deficiency: A risk factor for development of gestational diabetes mellitus**. *Diabetes & Metabolic Syndrome-Clinical Research & Reviews* 2019, **13**(2):985-987.

80. Telejko B, Kuzmicki M, Kretowska MZ, Szamatowicz J, Kretowski A: **A comparison of the International Association of Diabetes and Pregnancy Study Groups Recommendations with Former Criteria for Diagnosing Gestational Diabetes Mellitus: A Retrospective Cohort Study**. *Experimental and Clinical Endocrinology & Diabetes* 2019, **127**(6):359-366.

81. Wan CS, Abell S, Aroni R, Nankervis A, Boyle J, Teede H: **Ethnic differences in prevalence, risk factors, and perinatal outcomes of gestational diabetes mellitus: A comparison between immigrant ethnic Chinese women and Australian-born Caucasian women in Australia**. *Journal of Diabetes* 2019, **11**(10):809-817.

82. Wang Y, Zhang P, Chen X, Wu W, Feng Y, Yang H, Li M, Xie B, Guo P, Warren JL *et al*: **Multiple metal concentrations and gestational diabetes mellitus in Taiyuan, China**. *Chemosphere* 2019, **237**.

83. Yan B, Yu Y, Lin M, Li Z, Wang L, Huang P, Song H, Shi X, Yang S, Li X *et al*: **High, but stable, trend in the prevalence of gestational diabetes mellitus: A population-based study in Xiamen, China**. *Journal of Diabetes Investigation* 2019, **10**(5):1358-1364.

84. Yen IW, Lee CN, Lin MW, Fan KC, Wei JN, Chen KY, Chen SC, Tai YY, Kuo CH, Lin CH *et al*: **Overweight and obesity are associated with clustering of metabolic risk factors in early pregnancy and the risk of GDM**. *PLoS ONE* 2019, **14**(12).

85. Zahra S, Irum P, Bhatti SR: **SOCIAL & DEMOGRAPHIC RISK FACTORS OF DIABETES MELLITUS DURING GESTATION PERIOD**. *Indo American Journal of Pharmaceutical Sciences* 2019, **6**(5):10917-10922.

86. Zhang G, Wang X, Zhang X, Li Q, Xu S, Huang L, Zhang Y, Lin L, Gao D, Wu M *et al*: **Antimony in urine during early pregnancy correlates with increased risk of gestational diabetes mellitus: A prospective cohort study**. *Environment International* 2019, **123**:164-170.

87. Zhu B, Huang K, Yan S, Hao J, Zhu P, Chen Y, Ye A, Tao F: **VDR Variants rather than Early Pregnancy Vitamin D Concentrations Are Associated with the Risk of Gestational Diabetes: The Ma'anshan Birth Cohort (MABC) Study**. *Journal of Diabetes Research* 2019, **2019**.

88. Zhu B, Liang C, Xia X, Huang K, Yan S, Hao J, Zhu P, Gao H, Tao F: **Iron-Related Factors in Early Pregnancy and Subsequent Risk of Gestational Diabetes Mellitus: the Ma'anshan Birth Cohort (MABC) Study**. *Biological Trace Element Research* 2019, **191**(1):45-53.

89. Aburezq M, AlAlban F, Alabdulrazzaq M, Badr H: **Risk factors associated with gestational diabetes mellitus: The role of pregnancy-induced hypertension and physical inactivity**. *Pregnancy Hypertension* 2020, **22**:64-70.

90. Alsaedi SA, Altalhi AA, Nabrawi MF, Aldainy AA, Wali RM: **Prevalence and risk factors of gestational diabetes mellitus among pregnant patients visiting National Guard primary health care centers in Saudi Arabia**. *Saudi medical journal* 2020, **41**(2):144-150.

91. Bar-Zeev Y, Haile ZT, Chertok IA: **Association between Prenatal Smoking and Gestational Diabetes Mellitus**. *Obstetrics and Gynecology* 2020, **135**(1):91-99.

92. Basu J, Datta C, Chowdhury S, Mandal D, Mondal NK, Ghosh A: **Gestational Diabetes Mellitus in a Tertiary Care Hospital of Kolkata, India: Prevalence, Pathogenesis and Potential Disease Biomarkers**. *Experimental and Clinical Endocrinology and Diabetes* 2020, **128**(4):216-223.

93. Dos Santos PA, Madi JM, da Silva ER, Vergani DOP, de Araújo BF, Garcia RMR: **Diabetes gestacional na população atendida pelo sistema público de saúde no Brasil. Prevalência e fatores de risco**. *Revista brasileira de ginecologia e obstetricia : revista da Federacao Brasileira das Sociedades de Ginecologia e Obstetricia* 2020, **42**(1):12-18.

94. Francis EC, Li M, Hinkle SN, Cao Y, Chen J, Wu J, Zhu Y, Cao H, Kemper K, Rennert L *et al*: **Adipokines in early and mid-pregnancy and subsequent risk of gestational diabetes: a longitudinal study in a multiracial cohort**. *BMJ open diabetes research & care* 2020, **8**(1).

95. Ganapathy A, Holla R, Darshan BB, Kumar N, Kulkarni V, Unnikrishnan B, Thapar R, Mithra P, Kumar A: **Determinants of gestational diabetes mellitus: a hospital-based case–control study in coastal South India**. *International Journal of Diabetes in Developing Countries* 2020.

96. Kong M, Lu Z, Zhong C, Gao Q, Zhou X, Chen R, Xiong G, Hao L, Yang X, Yang N: **A higher level of total bile acid in early mid-pregnancy is associated with an increased risk of gestational diabetes mellitus: a prospective cohort study in Wuhan, China**. *Journal of Endocrinological Investigation* 2020, **43**(8):1097-1103.

97. Lan X, Zhang Y-q, Dong H-l, Zhang J, Zhou F-m, Bao Y-h, Zhao R-p, Cai C-j, Bai D, Pang X-x *et al*: **Excessive gestational weight gain in the first trimester is associated with risk of gestational diabetes mellitus: a prospective study from Southwest China**. *Public Health Nutrition* 2020, **23**(3):394-401.

98. Li X, Huang Y, Xing Y, Hu C, Zhang W, Tang Y, Su W, Huo X, Zhou A, Xia W *et al*: **Association of urinary cadmium, circulating fatty acids, and risk of gestational diabetes mellitus: A nested case-control study in China**. *Environment International* 2020, **137**.

99. Mishra S, Shetty A, Rao CR, Nayak S, Kamath A: **Risk factors for gestational diabetes mellitus: A prospective case-control study from coastal Karnataka**. *Clinical Epidemiology and Global Health* 2020.

100. Rayis DA, Musa IR, Al-Shafei AI, Moheldein AH, El-Gendy OA, Adam I: **High haemoglobin levels in early pregnancy and gestational diabetes mellitus among Sudanese women**. *Journal of obstetrics and gynaecology : the journal of the Institute of Obstetrics and Gynaecology* 2020:1-5.

101. Siddiqui K, George TP, Joy SS, Nawaz SS: **Association of sex hormone binding globulin with gestational age and parity in gestational diabetes mellitus**. *Journal of Maternal-Fetal and Neonatal Medicine* 2020.

102. Skupski DW: **Alphabet soup**. *BJOG* 2020, **127**(9):1153.

103. Yong HY, Shariff ZM, Yusof B-NM, Rejali Z, Appannah G, Bindels J, Tee YYS, van der Beek EM: **The association between dietary patterns before and in early pregnancy and the risk of gestational diabetes mellitus (GDM): Data from the Malaysian SECOST cohort**. *Plos One* 2020, **15**(1).
