# Supplementary figures and images for: Factors Associated with Gestational Diabetes Mellitus: A Meta-Analysis
Source: J Diabetes Res. 2021 May 10;2021:6692695. doi: 10.1155/2021/6692695 (PMC8128547; doi:10.1155/2021/6692695)

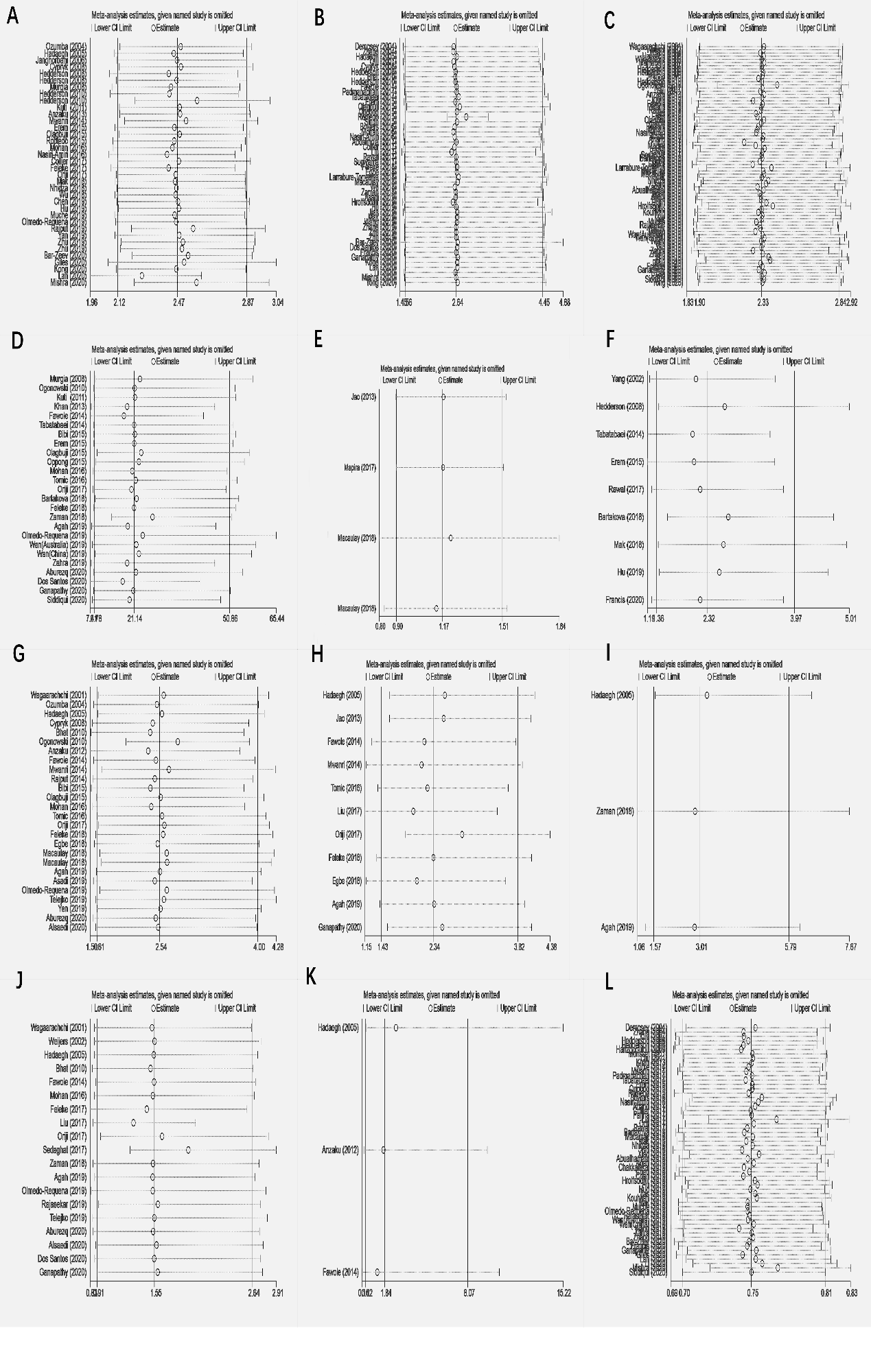

Supplement: Supplementary Materials — References of included studies. Sensitivity analysis: (A) maternal age ≥ 25 years; (B) prepregnancy overweight or obese; (C) FHD; (D) history of GDM; (E) HIV status; (F) pregestational smoking; (G) history of macrosomia; (H) history of stillbirth; (I) history of premature delivery; (J) history of abortion; (K) history of congenital anomaly; (L) primigravida. [file 6692695.f1.zip › 6692695.f2.docx]
